# Factors Influencing Excessive Dynamic Genu Valgum and the Effect on Post-Landing Movement Patterns: A Cross-Discipline Narrative Review

**DOI:** 10.3390/jfmk11010069

**Published:** 2026-02-08

**Authors:** Austin Granger, Akash J. Patel, Sammy K. Bonfim, Chamaree de Silva

**Affiliations:** 1 Macon Rehabilitation and Performance Center, Macon, GA 31210, USA; 2 Department of Biology, Mercer University, 1501 Mercer University Drive, Macon, GA 31207, USA; 3 Mercer Medical School, Mercer University, 1550 College Street, Macon, GA 31207, USA; 4 Department of Physics, Mercer University, 1501 Mercer University Drive, Macon, GA 31207, USA

**Keywords:** dynamic genu valgum, lower-limb biomechanics, landing, acceleration, deceleration, medial knee excursion, force attenuation, stretch–shortening cycle

## Abstract

This review summarizes the existing literature to investigate the role of excessive dynamic genu valgum (DGV) upon landing on subsequent movement performance in athletes. General systems theory and kinetic chain theory comprise the underlying theoretical frameworks, with an emphasis on regional interdependency in the context of lower-limb kinematics. Using a snowballing methodology, information was obtained from PubMed, CINAHL, Wiley Online Library, ProQuest, and Scopus databases, as well as through the utilization of Google Scholar and relevant biomechanics and movement analysis textbooks. Limitations include a paucity of research in the absence of injury and on DGV and subsequent performance post landing. Numerous factors, such as strength deficits of the predominant stabilizers of the knee in the frontal plane, fatigue, presence of dual tasks, and ingrained motor control, may influence medial knee excursion upon landing. Increased medial knee excursion during the transition from force attenuation to control is theorized to reduce the mechanical advantage of the quadriceps, impairing the efficiency of the stretch–shortening cycle for subsequent athletic movement performance. Mechanical and cognitive factors may influence knee biomechanics during landing and subsequent movement efficiency; however, the existing literature would benefit from further exploration of the differences in movement mechanics (e.g., acceleration) post landing in excessive DGV and the role of the trunk and subtalar joint on knee kinematics through the context of regional interdependency. This review is novel in investigating DGV from the perspective of movement performance rather than injury.

## 1. Introduction

Nearly all sports comprise some degree of running, jumping, or hopping; therefore, optimizing landing mechanics is beneficial for athletic performance [1,2,3,4,5,6,7,8]. Upon landing, muscle groups at multiple joints along the kinetic chain cooperate to mitigate excessive joint loading through effective attenuation of impact force. Deficits in effectively managing impact force may present via excessive dynamic genu valgum (DGV) [9,10,11]. Consistent with the principle of regional interdependency, DGV occurs as a multi-planar movement pattern; however, it is often considered a predominantly frontal plane movement. In the frontal plane, femoral adduction contributes to medial excursion of the tibiofemoral joint, eliciting a valgus Q-angle [9,12,13,14,15]. This medial excursion is coupled with femoral internal rotation and tibial external rotation in the transverse plane, alongside a degree of anterior tibial translation in the sagittal plane [1,10,14]. Many individuals present with a slightly valgus Q-angle; however, this study more so highlights the ability to manage reactionary force from the ground upon landing, with observable changes in frontal plane deviation from rest being representative of functional stability [13,14,16,17,18,19,20]. According to Herrington and Munro [13], excessive DGV can be considered greater than 9 degrees in males and 12 degrees in females in a single-leg drop test. The relative increase in DGV in females is postulated to be a contributing factor to the higher prevalence of anterior cruciate ligament (ACL) tears, which comprises a majority of research on DGV [21,22,23]. This review is novel in its emphasis on motor performance, with DGV likely altering the efficiency of the stretch–shortening cycle (SSC), impairing subsequent force production requiring a longer time to stabilization, and challenging reactive postural stability [8,24,25,26,27,28,29]. The SSC supports that eccentric load of the gluteal musculature, quadriceps, ankle plantarflexors, and more at the attenuation phase facilitates the storage of elastic energy that will facilitate subsequent forceful concentric contractions; impaired eccentric control may disrupt movement mechanics [8,24,30,31]. Therefore, a review of DGV in the context of landing mechanics necessitates an understanding of multi-joint and multiplanar kinematics to elucidate implications on optimizing movement efficiency and athletic longevity [4,5,8,9,10,11,22,23,24,25,28,29,32].

## 2. Theoretical Frameworks

This review operates within two theoretical frameworks: general systems theory and kinetic chain theory. General systems theory posits that understanding any phenomenon necessitates investigation into the interactions of subsystems rather than each subsystem in isolation [33]. For instance, landing requires effective neuromuscular control for effective distribution of the resulting ground reaction force. The ability to effectively attenuate force may be altered in the presence of various other psychosocial phenomena; therefore, an interdependency may exist between cognitive demands and performance [34,35,36,37,38]. Cognitive load functions as an interacting subsystem that can influence sensorimotor integration and modify neuromuscular strategies used to control landing mechanics [34,35,38,39]. The interdependency of subsystems seen in general systems theory aligns with the premise that DGV is not solely a knee phenomenon but the result of multi-joint coordination patterns influenced by both biomechanical and cognitive subsystems [4,6,10,11,33,34,35,38,39].

This framework fits well with kinetic chain theory to elucidate how frontal plane stability at the knee is influenced by trunk, hip, knee, and foot kinematics; inefficiencies above or below the knee may significantly affect the force placed upon the knee [4,6,10,11,18,19,40,41,42,43]. Therefore, DGV may be facilitated by ineffective force accommodation proximal or distal to the knee [2,16,18,38,44,45,46]. The resulting medial knee excursion may alter the quadriceps vector angle, potentially impairing the efficiency of the SSC and hindering force production for subsequent movement [8,25,29]. Therefore, the basis for understanding the implications of DGV upon subsequent movement performance after landing is general systems theory and kinetic chain theory, emphasizing the integration of multi-joint neuromuscular and sensorimotor contributions to effective force attenuation and control [33,40].

## 3. Materials and Methods

A narrative review approach was selected to elucidate the factors influencing excessive DGV and the implications on movement performance due to a lack of extensive experimental research directly linking DGV to post-landing movement patterns. This review offers an integrative, cross-disciplinary synthesis of the literature that includes lower-limb biomechanics, performance characteristics (e.g., explosiveness), neuromotor control and motor feedback, the cognitive demands during sport performance, and more.

A literature search was performed by A.G. between 1 June 2025, and 30 January 2026, using PubMed, CINAHL, Wiley Online Library, ProQuest, and Scopus databases, alongside the utilization of Google Scholar and relevant biomechanics and movement analysis textbooks. Search terms and key concepts included “dynamic genu valgum,” “landing biomechanics,” “medial knee displacement,” “knee valgus torque,” “ground reaction force,” “shock absorption,” “rate of force development,” “stretch-shortening cycle,” “postural sway,” “proprioception,” “neuromotor control,” “kinetic chain,” “hip abduction,” “hip extension,” “hip external rotation,” “knee extension,” “knee flexion angles,” “range-of-motion,” “sagittal plane mechanics,” “frontal plane mechanics,” “shock absorption,” “performance,” “balance,” “dual task,” and “motor control.” No time constraint was placed on the publication date of the articles.

Articles were eligible for inclusion if they were peer-reviewed and yielded information on DGV, landing mechanics, or the functional implications of DGV within a systems-based approach or through a model of regional interdependency. Studies that investigated DGV in the context of injury were included if findings could plausibly inform movement performance or force attenuation. Non-peer-reviewed studies, as well as those studying non-human models, were excluded. Titles and abstracts of the article list yielded by the above search terms were first screened for inclusion. After identifying key articles, a snowballing search methodology was employed by examining the reference lists of included studies to improve the comprehensiveness of the review. Articles found via the snowballing methodology also met the inclusion criteria.

This study aims to discuss the performance implications of DGV upon landing, resulting in research objectives that include summarizing the kinetic chain approach to DGV, implications of DGV on force attenuation in landing, implications of DGV on explosiveness and SSC, how DGV may influence balance, and the relationship between dual task and DGV. However, this study has limitations. Limitations include a lack of research directly investigating performance associated with DGV outside of knee injury and a larger evidence base pertaining to the female athlete population. Additionally, this article cannot make causal claims, secondary to the lack of experimentation.

## 4. Lower-Limb Kinematics

DGV may occur secondary to an inability to control external moments influencing knee position upon landing. The application of force by the foot onto the ground results in an equal and opposite ground reaction force (GRF), consistent with Newton’s third law of motion [3,14,47,48]. A high magnitude GRF vector upon landing correlates directly to joint loading, and this value may vary throughout landing as the athlete attempts to control deceleration [6,49,50,51,52]. The magnitude of the GRF vector is the sum of its three orthogonal components: vertical, anterior–posterior (AP), and mediolateral. DGV occurs predominantly in the mediolateral axis [14,47]. However, research on landing mechanics appears to emphasize the vertical GRF to a greater extent; poor force attenuation from landing likely facilitates aberrant and compensatory lower-limb kinematics, such as DGV [4,6,11,19,41,42,43,53]. Understanding force management in the other orthogonal components is necessary to understand movement patterns in DGV occurring outside of the frontal plane and the overarching changes in the GRF vector [4,5,7,9,14,25,28,29,41,42,43,54,55].

In the context of the vertical GRF, Harry and colleagues [6] note that landing comprises three phases: loading, attenuation, and control. The loading phase refers to the time between initial contact and maximum magnitude of the vertical GRF [6]. Landing from a single-leg jump would result in a more pronounced impact peak than walking or running, necessitating effective dynamic management of the vertical GRF [52,56]. Vertical GRF is then reduced in the attenuation phase, largely due to numerous factors that include but are not limited to eccentric quadricep and gluteal control [6,9,10,11,17,23,24,57,58,59,60,61]. After full attenuation, the vertical GRF rises again during the control phase as the athlete generates force through their foot into the ground for preparation for subsequent movement, ending the landing phase [6]. A similar phenomenon occurs in walking, speed-walking, and running, where the vertical GRF appears to increase at varying extents at initial contact and again at push-off [56]. In the AP axis and throughout landing from a jump or hop, the posterior chain activates to mitigate forward trunk lean to maintain balance. In the context of DGV, anterior tibial translation may be limited by activation of the hamstring and gastrocnemius, and research suggests that optimizing co-activation upon landing, alongside an increased knee flexion angle, may be beneficial in improving landing mechanics for both subsequent performance and to ameliorate injury risk [11,19,38,62,63,64].

Mediolateral GRF with a direction lateral to the midpoint of the knee will contribute to DGV secondary to the production of a valgus torque. This torque can be influenced significantly by inert and dynamic systems of stability at, above, and below the knee joint [4,5,7,9,14,25,28,29,41,42,43,54,55]. For instance, the medial patellofemoral ligament is an inert structure acting to limit lateral patellar displacement relative to the femur, limiting patellofemoral maltracking [65]. In DGV, accompanying lateral tracking of the patella may affect the force vector elicited by the quadriceps and hinder subsequent force production, influencing dynamic stability. Analyzing DGV becomes much more intricate when examining dynamic contributions across multiple joints for mitigating valgus knee torque [4,5,7,9,25,28,29,41,54]. Therefore, this review emphasizes systems and kinetic chain theory to evaluate the neuromuscular contributions at each joint independently before discussing their overall contributions to lower-limb kinematics during the landing phase [66,67].

### 4.1. Hip

The hip allows triplanar movement patterns, with frontal plane moment arms being most significant for the adductor brevis, adductor longus, the anterior head of the adductor magnus, the anterior and middle fibers of the gluteus medius and minimus, the gracilis, and the tensor fascia latae (TFL). Some muscles that largely operate in the sagittal plane may contribute to frontal plane movement mechanics. For instance, the rectus femoris likely has an approximately 2.3 cm moment arm for abduction (versus a 4.3 cm moment arm for hip flexion) [57]. The emphasis on mitigating medial knee displacement necessitates optimal eccentric control of the primary hip abductors (e.g., gluteus medius, gluteus minimus, TFL) and external rotators (gluteus maximus, obturator internus, gemellus superior, gemellus inferior, and quadratus femoris) to mitigate the femoral adduction and internal rotation seen in DGV. Eccentric neuromotor control of these muscles, alongside the range of motion of hip internal rotation, significantly influences medial knee displacement during landing [10,17,20,23,57,58,59,60,61,68].

Alterations in hip abduction recruitment influence knee position during landing [23,69]. Peak rate of torque development (RTD) of the hip abductor musculature is a metric referring to explosiveness and is postulated to minimize peak DGV; Stearns-Reider and colleagues [23] evaluated RTD and peak DGV during landing among females, finding that peak RTD in the first 50 milliseconds after foot contact was significantly correlated with the extent of DGV. However, this correlation was not significant in the first 200 milliseconds after foot contact, potentially secondary to variability in how athletes may attenuate and control the magnitude of the GRF vector upon landing [6,23]. These findings are supported by numerous other studies indicating that hip abductor strength and DGV are likely inversely correlated [10,17,20,27,41,70]. Similarly, repeated jumping results in reduced hip abduction moments in both double and single leg landing; therefore, DGV may increase upon repeated attempts secondary to reduced eccentric force production of the hip abductors [41]. The ability to mitigate compensatory valgum upon landing may be reduced in the presence of significant hip abductor fatigue, secondary to reduced functional capacity to perform eccentric work. Similarly, asymmetric fatigue of the pelvic girdle can disrupt proximal stability and increase the mechanical demand of the affected knee [41]. Impaired stability of the pelvic girdle and lower extremity is likely not occurring secondary to structural pelvic asymmetries in most athletes; however, the presence of severe fatigue may result in stiffer landings [71,72,73]. Contemporary evidence suggests that increased hip flexion upon landing is necessary for force attenuation [43,74]. Therefore, managing fatigue may be a significant method of ameliorating performance deficits associated with DGV later in a competitive event [20,75]. This is consistent with research by Lagouvardou and colleagues [75], who argue that the endurance of the hip abductors is a more significant factor than strength in improving valgus control. Similarly, Carcia and colleagues [20] found that the degree of DGV increased with fatigue despite an unchanged vertical GRF during performance of the drop jump test, alongside a significant reduction in hip abductor force generation. They postulate that compensatory mechanisms likely contribute to the unchanged vertical GRF; however, the GRF in the frontal plane is likely significantly influenced by muscular capacity for eccentric work, facilitating compensatory hip adduction [20].

Secondary to an influence on the vertical GRF, deficits in fatigue and neuromuscular control outside of the frontal plane may contribute to DGV through altered energy absorption of the closed kinetic chain [19,43]. One cross-sectional study examining 28 female youth athletes suggested that decreased mechanical energy absorption of the hip extensors may be correlated with increased DGV at the total landing phase [19]. Per the phases described by Harry and colleagues [6], eccentric hip extension in the control phase counters an external hip flexion moment, with reduced work done by the hip extensors potentially contributing to DGV [19,43]. In the sagittal plane, fatigue was reported to result in changes to a reduction in hip flexion angles upon landing, resulting in a reduction in force absorption that likely facilitates valgus torque at the knee [43]. The hamstring musculature contributes by helping control hip flexion moments through a primary action of hip extension eccentrically during force attenuation [57,70].

In the transverse plane, research suggests that excessive internal rotation range-of-motion (ROM) and strength deficits of the hip external rotators (i.e., predominantly gluteus maximus) contribute to DGV, and these variables likely differ significantly between sexes [17,58,59,61,68]. An experimental, cross-sectional study by Hodel and colleagues [58] examining male and female competitive soccer players found statistically significant baseline increases in hip internal rotation and a significant baseline reduction in max eccentric and concentric peak force generated by the hip external rotators among females. This sample then performed drop jump landings before and after fatiguing activity, with the female group having a statistically significant positive relationship between hip internal rotation and medial knee displacement upon landing (consistent with DGV); however, the male group did not have a statistically significant relationship [58]. Females may have a reduction in inert stability secondary to relative joint laxity versus males, and the relative joint laxity compounded with reduced capacity for repetitive eccentric work of the hip external rotators likely contributes to sex-specific changes in DGV upon landing in a fatigued state [58,59,61,68]. Among collegiate female athletes, Malloy and colleagues [59] found that hip external rotator strength was statistically associated with reduced knee excursion in the transverse plane and increased femoral adduction during unexpected single-leg landing tasks. The increase in frontal plane excursion is theorized to be secondary to adaptive movement patterns, where continuous stimulation of the hip external rotators in landing mechanics associated with DGV may result in increased strength [59,76]. Findings by Llurda-Almuzara and colleagues [32] support this hypothesis, suggesting that motor control and activity of the central nervous system may be specifically beneficial in mitigating DGV. Therefore, the relationship between hip external rotator strength and DGV may be multifaceted, influenced by ingrained functional movement patterns and the capacity of the external rotators to perform eccentric work [17,32,58,59,61,68,76].

However, not all research supports that the strength of the hip abductors, extensors, and external rotators is consistently associated with DGV [46,77]. A 2021 systematic review by Alzahrani and colleagues [77] showed inconsistent findings relating hip strength to DGV, specifically during a single-leg step down, single-leg squat, or forward lunge. However, the vertical GRF upon landing is higher, requiring greater force production from the muscles that provide dynamic stability to limit excessive femoral adduction and internal rotation [77]. A systematic review by Dix and colleagues [46] finds a statistically significant association between DGV and hip abduction, extension, and external rotation strength in single-leg landing tasks among active women. However, this association was not significant during double-leg landing tasks, potentially due to a more evenly distributed load that necessitates less stabilizing forces than that of single-leg landing [46]. Inconsistent research likely exists because, from a systems approach, strength is not a sole determinant of functional movement patterns. As mentioned above, endurance plays a significant role in repeated activities [20,58]. Additionally, an individual’s practice with a specific movement with associated motor control development regarding the hip may also facilitate varying knee landing mechanics, regardless of functional hip strength or endurance [32]. Therefore, the law of specificity in strength and conditioning should apply to allow athletes to not only strengthen and condition relevant hip musculature but also practice the specific movement demands required in sport [1,2,3,10,20,32,78].

Dynamic systems at the hip being a predominant influence on DGV is supported by numerous studies highlighting that hip abductor, extensor, and external rotator weakness may be correlated to the extent of DGV, especially in single-leg ballistic tasks [9,10,19,20,41,43,46,59]. Extensive training that emphasizes strength and eccentric control is postulated to reduce DGV [1,2,78]. For instance, Mozafaripour and colleagues [78] utilized an 8-week corrective exercise program among young male athletes and found that the experimental group had significant reductions in DGV during a single-leg squat with increased hip abductor and external rotator muscle strength versus a control group. Similarly, a review by Wilczyński and colleagues [10] suggests that the incorporation of biofeedback for the hip abductors, extensors, and external rotators may be effective at ameliorating DGV during single-leg landing. However, neuromuscular fatigue plays a significant role in the functional capacity of the muscles that work to mitigate DGV, warranting endurance training of the relevant hip musculature [78]. Additionally, athletes may benefit from specific intervention targeting neuromotor control of sport-specific tasks to improve landing mechanics through permitting increased hip flexion for appropriate shock absorption [2,79]. Therefore, the hip may be a target for intervention for reducing DGV, with inefficiencies in force attenuation and control during landing likely impairing subsequent motor performance [1,2,78,79].

### 4.2. Knee

Attenuating force upon landing necessitates appropriate dynamic neuromotor control of the musculature of the knee [16,80,81,82,83]. The knee comprises the tibiofemoral and patellofemoral joints, and the quadriceps act directly on both. The quadriceps tendon has a tri-laminar structure comprising a superficial layer (rectus femoris), a deep layer (vastus intermedius), and an intermediate layer (vastus lateralis and vastus medialis) [84]. While considered a muscle group that predominantly acts in the sagittal plane, Claiborne and colleagues [16] note that the functional strength of the quadriceps and hamstrings likely accounts for approximately 13.6% and 18.1% of variability in predicting frontal plane movements, respectively. For instance, the vastus medialis may contribute to the knee abduction moment through medial insertion into each of the three layers of the quadriceps tendon and a convergence with the medial patellofemoral ligament. For this reason, the vastus medialis is postulated to significantly influence patellofemoral mechanics in DGV [54,84]. The combination of femoral adduction and tibial abduction facilitates lateral patellar tracking, and the vastus medialis is postulated to be a significant contributor to patellofemoral stability. Understanding patellar tracking during DGV is necessary secondary to its implications on quadriceps force production. Increased lateral tracking of the patella accompanying medial knee excursion upon landing influences the orientation of the quadriceps vector, potentially reducing its effectiveness at managing the vertical GRF [25,28,29]. Additionally, the vastus medialis may be in a disadvantageous position versus the vastus lateralis and reduce shock absorption, facilitating DGV [80,81,82]. A surface electromyographic (sEMG) study by Park and colleagues [80] found statistically larger vastus lateralis than vastus medialis activity at isometric testing at 30 and 60 degrees of knee flexion in a group with static genu valgum versus static genu varum and control groups in a standing position, suggesting a relationship between vastus medialis and lateralis activity and frontal plane mechanics. These findings are consistent with those found by Park and colleagues [81], who found significantly faster onset time of the vastus lateralis versus the vastus medialis in stair ascent and descent. Additionally, higher vastus lateralis and lower vastus medialis pre-activity activation is associated with increased DGV angles [82]. Variation in inter and intra-limb (quadricep or hamstring) asymmetry in tone, mass, and strength is often present in athletes and may influence neuromuscular recruitment [85,86,87,88,89,90]. In functional activities that necessitate eccentric quadricep neuromotor control, vastus lateralis and vastus medialis recruitment activity are likely higher in individuals with DGV, secondary to the need for dynamic stability of the knee [83].

Fundamentally, landing requires significant eccentric control of the quadriceps to attenuate force [19,91,92]. Mechanical energy absorption (MEA) of the knee extensors is significantly correlated with an increase in DGV during both single-leg and double-leg landing tasks [19,92]. MEA patterns among females and males may be different, and some researchers postulate that these changes may be secondary to an increased propensity of female athletes to use their quadriceps to dynamically manage force upon landing, with a contributing factor likely being the significant differences in gluteal force production in females versus males [27,93]. Females also have an increased Q-angle versus males, which may alter the force vector of the quadriceps to most efficiently handle the GRF [25,28,29,93]. These findings likely correlate with women having twice the knee valgus angular velocity during landing than men [94]. Deficits in eccentric motor control in the sagittal plane may have ramifications in frontal plane mechanics, resulting in DGV secondary to compensatory movement patterns.

The co-contraction ratio of the quadriceps and hamstrings may influence the dynamic stability of the knee upon landing [11,63]. Specifically, an emphasis on the biceps femoris in DGV seems prevalent in the literature. The long and short heads of the biceps femoris merge to insert on the lateral tibial condyle and fibular head; therefore, the biceps femoris may contribute in the transverse plane to tibial external rotation. Likewise, the biceps femoris activation seen in landing tasks may contribute to DGV [10,63,65]. Investigating single-leg landing tasks in badminton, Hu and colleagues [63] find that the lateral hamstring to quadricep co-contraction ratio was significantly and positively correlated to an increased valgus moment at the knee. Pre-activation of the vastus lateralis and biceps femoris has been found to be associated with increased DGV angle during landing tasks; however, these findings were not consistent with a study investigating knee musculature pre-activation with sEMG [10,82,95]. Similar to that of the relationship of the vastus medialis and vastus lateralis, the semitendinosus is postulated to counteract the valgus moment facilitated by the biceps femoris [83]. However, a paucity of research exists investigating semitendinosus strength or pre-activation with DGV in single-leg landing tasks. Ultimately, medial to lateral quadricep and hamstring relationships appear analogous, with notable asymmetries potentially facilitating aberrant frontal plane mechanics [80,81,82,83].

Knee flexion angles upon landing influence the magnitude of the vertical GRF and the moment arm of the quadriceps. Decreased knee flexion angles at the loading phase correspond with greater vertical GRFs, necessitating increased reliance on dynamic stability and increased propensity for aberrant and compensatory movement mechanics [11,64]. Additionally, increased knee flexion angles are significantly correlated with increased extensor moment arms [11]. A larger moment arm requires less force to produce the same torque, effectively improving the mechanical advantage of the quadriceps [3,96]. Therefore, increased knee flexion upon the loading and attenuation phases results in increased management of the vertical GRF, limiting compensatory valgus torque at the knee [11]. Reduced quadricep mechanical advantage and deficits in eccentric control for effective force attenuation may influence the time to stabilization for subsequent activity and concentric force generation out of the attenuation phase [11,19,26,91,92].

### 4.3. Foot and Ankle

Secondary to regional interdependency, the talocrural and subtalar joints contribute to managing the propagation of force up the kinetic chain upon landing, with initial contact likely resulting in a force more than thirteen times body weight [97]. Specifically, foot external rotation and ankle eversion are attributable to the movement pattern seen in DGV [98,99]. Foot positioning upon landing may influence the angle of the resulting GRF vector. A study by Teng and colleagues [99] showed that the knee valgus moment increased near the beginning and approximately 60% into the landing phase among male recreational basketball players in various foot transverse plane orientations (inward rotation, forward, and outward rotation). However, landing in a toe-out position resulted in a significantly greater knee valgus moment than the other tested positions [99]. An outward toe position upon landing may accompany external rotation of the foot and eversion of the ankle, facilitating tibial external rotation that contributes to increased valgus torque at the knee [98,99,100]. However, there appears to be a paucity of evidence on foot positioning during landing in relation to DGV; therefore, further research is warranted. The valgus torque produced at the knee may also be influenced by variations in foot and ankle ROM, alongside relevant muscular strength and endurance [10,44,45,101,102,103].

Initial contact and force attenuation are coupled with eccentric plantarflexion as the ankle moves into dorsiflexion. Similar to how increased hip and knee flexion angles upon landing allow for improved shock absorption, optimal landing mechanics necessitate sufficient foot and ankle ROM [11,101]. The contemporary literature suggests that ankle dorsiflexion ROM is especially important in reducing shock absorption upon landing, secondary to its implications on knee flexion angle and the magnitude of the vertical GRF [101]. In closed kinetic chain (CKC), the tibia glides anteriorly over the talus; therefore, reduced CKC dorsiflexion impairs forward tibial advancement and reduces the capacity for an athlete to land with an increased knee flexion angle [14,65,104]. These implications of ankle dorsiflexion ROM on DGV are supported by a systematic review and meta-analysis published by Lima and colleagues [101], who argue that talocrural mobility has significant implications on lower-limb kinematics. Consequently, the reduced knee flexion angle increases the magnitude of the vertical GRF and contributes directly to the compensatory mechanisms that facilitate DGV [11].

The subtalar and talocrural joints may significantly influence DGV upon landing [10,44,45,53,97,102,103]. Interestingly, one research article argues that foot pronation may not significantly influence the vertical GRF; however, the use of the navicular drop test to assess functional foot pronation is valid in gait mechanics but may not be generalizable to landing mechanics [53]. Generally, the subtalar responses to landing activities are not well described in the contemporary literature, making this an avenue for potential research. Nevertheless, the talocrural joint likely absorbs the most shock in landing (likely greater than 80%), with a reduction in peak loading rate correlating with increased plantarflexion angles in the loading phase [97,102]. The dorsiflexion coupled with calcaneal eversion seen in foot pronation results in a higher load to the lateral facet, potentially shifting the angulation of the GRF vector more laterally to the patellar midpoint [97]. Consequently, impairments in dorsiflexion ROM in CKC may result in compensatory movement mechanics in other joints. For instance, limited dorsiflexion ROM likely contributes to greater hip adduction and subtalar eversion, influencing landing mechanics in a manner that may increase valgus torque at the knee [10,44,45,103].

Because of a significant external dorsiflexion moment upon landing, force attenuation necessitates significant eccentric strength of the ankle plantarflexors, similar to eccentric quadriceps strength in decelerating knee flexion [53,91]. For this reason, the eccentric function of the plantarflexors and quadriceps is significant for the deceleration seen in the force attenuation phase [65,91,105]. The gastrocnemius and soleus are both powerful ankle plantarflexors, and their contributions to this moment vary based on knee angle [65,106]. With increased knee flexion, plantarflexion torque reduces secondary to a mechanical disadvantage of the gastrocnemius, secondary to its origin superior to the knee joint line. Consequently, the soleus is a significant synergist in attenuating force and mitigating the anterior tibial translation seen in DGV [11]. The tibialis posterior is another plantarflexor that also contributes in the frontal plane to produce inversion concentrically and resist eversion eccentrically [65,107]. Komatsu and colleagues [108] argue that landing in an inverted position improves the lever arm for the ankle plantarflexors through increasing foot rigidity. They find that fatigue of the inverters (e.g., posterior tibialis secondary to its significant implications on resisting CKC pronation) results in increased work demands of the superior joints when landing in a more medial position, suggesting decreased capacity of the inverters to perform the eccentric work that mitigates significantly increased foot pronation [108]. The knee and hip joints incur more shock, likely secondary to a reduced mechanical efficiency of the plantarflexors to effectively attenuate the vertical GRF [11,105,108]. Through a similar dynamic control logic to gait mechanics, coordinated activation of the musculature at the ankle, hip, knee, and trunk regulates angular momentum during landing. With the hip extensors predominantly opposing forward trunk rotation, the knee and ankle extensors modulate the magnitude and timing of angular impulse during force attenuation. Stability in landing occurs when this combined angular impulse stays within a controlled range [6,11,109,110]. Because of regional interdependency, the ability of the plantarflexors to mitigate force propagation up the kinetic chain is influenced by distal foot mechanics and directly affects more superior limb kinematics [108].

Proprioceptive sense may play a significant role in how athletes land; however, there is a paucity of experimental trials investigating these variables in DGV directly. Afferent nerves from proprioceptors provide information regarding joint position and kinesthetic sense [14,111]. These sensory afferents may facilitate neuroplastic changes that have significant implications for neuromotor control [111,112]. Individuals with proprioceptive deficits have increased postural sway during single-leg stance, and DGV is commonly associated with deficits in postural control [22,112]. While both proprioceptive impairment and DGV may result in relative deficits in functional stability, more research is required to correlate the two variables. Ultimately, deficits in distal force attenuation secondary to positioning, dorsiflexion ROM deficits, eccentric plantarflexion strength deficits, and proprioception may deleteriously influence the transition from force attenuation to force generation secondary to lower-limb aberrant movement mechanics, impeding post-landing movement performance [22,45,99,105,106,108,112].

### 4.4. Trunk

Musculature of the trunk likely influences lower-limb kinematics upon landing, facilitating DGV [42,113]. One study found that increased trunk flexion correlated significantly with a reduction in vertical GRF [42]. This finding is similar to those concerning the hip, knee, and ankle, where less erect posturing significantly improves shock absorption [5,10,11,43,44,45,103]. Consequently, a flexed trunk upon landing results in reduced quadriceps work, with Blackburn and Padua [42] finding a significant reduction in quadriceps activity during drop-landing tasks. Additionally, the gluteus medius and quadratus lumborum co-contract for frontal plane stability upon landing. Weakness of these muscles may be evident with a compensatory ipsilateral hip hike (i.e., Trendelenburg), inducing relative trunk sidebending towards the affected leg [10,65,113]. Consequently, Wilczyński and colleagues [10] suggest that improving the strength and neuromotor control of the hip abductors and trunk lateral flexors may play a significant role in preventing valgus collapse at the knee. The importance of trunk motor control on dynamic stability of the leg in CKC is supported by other scientific literature that finds deficits in neuromuscular recruitment associated with increased injury risk for female athletes [114,115]. There is a gap in the contemporary literature examining trunk motor control on DGV, specifically in the transverse plane. Further literature investigating the eccentric strength of the paraspinals in force attenuation (similar to that of the gluteals and quadriceps), the co-contraction ratios of the left versus right trunk lateral flexors and rotators, and the co-contraction ratio of the quadratus lumborum and gluteus medius during single-leg landing will be beneficial to elucidate training protocols through a framework of regional interdependency. Regarding movement performance, shock absorption may be attenuated by increased forward trunk flexion, allowing for improved time to stabilization and transition to concentric work of the lower extremity [10,42,113,114,115].

The above sections elucidate potential research opportunities in lower-limb biomechanics and movement performance. Secondary to multi-joint interdependency, researchers may benefit from investigating the impact of DGV on force generation of the gluteals, quadriceps, and plantarflexors post landing, the role of the semitendinosus in frontal plane knee stability, the role of the subtalar joint and its contributions to more proximal stability, the implications of proprioception on landing mechanics, and the role of the trunk on DGV regarding force generation out of a landing position. These gaps are highlighted below in Table 1.

## 5. Implications on Performance

Excessive DGV upon landing may influence how athletes attenuate force, influencing subsequent movement performance and increasing reactive postural stability demands. These demands will inherently differ based on how the athlete lands; one study found that the GRF impulse upon landing was approximately 1.6 times more when landing on one leg [116]. A double-leg landing allows for sharing of the vertical GRF between each contacting limb, reducing the demands for functional stability. However, staggered landings may be considered a double-leg landing but have an asymmetry of load. When performing a single-leg landing, the contacting limb must effectively absorb all of the available force to resist aberrant lower-limb kinematics [3]. When an athlete decelerates, the body undergoes repetitive single-leg landings; therefore, DGV may influence the efficiency of deceleration secondary to impairing the mechanical advantage of the quadriceps and gluteal musculature and potentially increasing time to stabilization [25,26,27,28,29,117]. Coordination among muscle groups that effectively offset one another’s destabilizing effects leads to balance during landing tasks. A backward angular momentum is generally produced by gravity and hip and knee extensors in initial contact. The effectiveness of co-contraction ensures stability in movement when the combined angular impulse stays within a controlled range [109]. Consequently, each landing type necessitates adequate motor control of numerous muscle groups throughout the kinetic chain to optimize multiplanar stability upon initial contact to force attenuation and control. The functional capacity and coordination of these muscle groups may be hindered in the presence of fatigue, potentially inducing an increase in valgus knee torque [20,75]. Within the above context of regional interdependency, this section investigates how DGV upon landing may influence numerous facets of performance, as well as how the competitive environment may facilitate DGV versus a training environment.

### 5.1. Implications of Shock Absorption on Subsequent Movement Performance

Athletes with greater capacity to attenuate force upon landing may have improved landing performance, and stiffness in the kinetic chain may result in a reduced capacity to effectively absorb the resulting vertical GRF, potentially impairing subsequent movement [4,5,6,7]. A study by Ishida and colleagues [7] found that females have a statistically increased knee abduction angle in double-leg landing with a subsequent jump versus without, and this angle markedly increased 45 milliseconds after initial contact (after peak knee flexion angle). In research by Harry and colleagues [6], roughly 45 milliseconds corresponds with the end of joint loading, just before maximum vertical GRF and transition to the attenuation phase of landing. Ishida and colleagues [7] also investigated males but did not find a statistically significant difference in knee abduction angle; therefore, female athletes may have more difficulty transitioning into the attenuation phase of landing versus male athletes. These findings are consistent with a study by Bates and colleagues [5], where adolescent female athletes had significant reductions in knee and hip flexion angles in the second landing, facilitating a marked increase in hip adduction and knee abduction angles. This is consistent with Ambegaonkar and colleagues [4], who found increased vertical GRFs in second landings versus first landings (i.e., drop jumps versus drop landings) among 15 physically active females. Another study with 30 female participants found statistically significant peak knee abduction moments with a similar vertical GRF in single leg landings with versus without a subsequent jump; the group performing the subsequent jump also had statistically significant trunk lateral tilt and rotation angles with increased hip internal rotation, facilitated by a center of gravity more medial than the base of support [41]. Ultimately, female athletes seem to have a more difficult time attenuating force than male athletes, likely secondary to a combination of neuromotor control and functional strength [118,119].

This difficulty may correlate with a reduced rate of force development (RFD), a metric associated with explosive movements [120]. There is a significant paucity of research investigating landing mechanics and subsequent RFD. However, one study investigating drop jumping on sand noted a reduction in peak vertical GRF with increased knee flexion angles and RFD [121]. Similarly, other studies suggest that landing tasks on sand may be a more beneficial starting point versus firm ground for rehabilitation programs (i.e., including ACL repairs) secondary to a lower vertical GRF, reduced medial knee excursion, and reduced knee shear force for females [122,123]. While studies emphasizing landing strategies on sand may not be a perfect correlate with all athletic sports, the sand being a softer surface may provide insight into the relationship between peak vertical GRF and RFD. Other studies have noted increased DGV when landing on inclined and wet surfaces [124,125].

An athlete with valgus collapse may have difficulty in subsequent force generation due to the SSC [8,24,30,31]. Because joint loading directly influences movement patterns, subsequent RFD may be significantly influenced by the reduced efficiency of the SSC apparent in DGV associated with stiffer landings [8,24,126]. Through the principle of specificity, an emphasis on eccentric training to improve RFD may be beneficial [127]. Theoretically, DGV may influence acceleration, change-of-direction, and subsequent jumping through impaired propulsion from a less efficient SSC; however, research on these topics is minimal and not conclusive [128,129].

DGV may hinder jump height and facilitate longer ground contact times [9,130,131]. Dewald and colleagues [9] suggest that individuals with DGV upon landing may be correlated with increases in ground contact time, reducing the reactive strength index (RSI); they postulate that reducing contact time may be an adequate cue for ameliorating knee abduction angle in DGV. The RSI provides significant information regarding an individual’s ability to transition from an eccentric contraction to a concentric contraction (i.e., transition from the attenuation to control phase of landing) with implications on reactive strength and RFD during plyometric movements [6,132]. One study found that an exercise program for DGV resulted in increased vertical jump height; however, conflicting articles exist correlating DGV and vertical jump height [9,118,119,130]. Ultimately, the literature seems to suggest that women have less strength and motor control of the key stabilizing musculature to ameliorate DGV upon landing, with potential implications for subsequent jumping [118,119]. This finding may suggest that females may benefit from a particular emphasis in strength and conditioning programs to improve force attenuation via strengthening of dynamic stabilizers to control for excess DGV. This emphasis may improve not only performance but also athletic longevity, as female athletes are at a significantly higher risk of ACL tear secondary to non-contact injuries often associated with DGV upon jump landing [21,22,23].

### 5.2. Postural Sway and Balance

Landing in DGV may influence the dynamic balance needed for subsequent force generation. Females with increased knee abduction angles during single-leg landing have been found to have significantly increased variability in foot positioning at initial contact, implying motor control deficits in dynamically adapting to various external demands [22]. Proprioceptive deficits are likely a notable contributing factor to facilitating a higher variability of landing biomechanics, secondary to the role of proprioception in joint position sense. Interestingly, increased joint laxity may correspond with increased proprioceptive deficits, likely a contributing factor to disproportionate ACL injury in female athletes versus males [74]. Similar to strength and endurance, proprioception also worsens with fatiguing activity, increasing aberrant lower-limb kinematics secondary to increased reliance on dynamic stabilizers [74,133]. From the context of regional interdependency, the proprioception at the ankle joint should contribute to alterations in knee abduction angle. Nevertheless, pressure on the sole of the foot during landing is likely the most significant factor influencing proprioception, but the combination of multi-joint proprioceptive input may directly influence postural stability [134]. Similarly, athletes returning from injury (e.g., ankle sprains, ACL injuries) may have impaired proprioception at the ankle and knee joints versus pre-injury [135,136,137]. Reduced lower-limb position sense upon landing with subsequent change in direction, jumping, or forward acceleration may result in aberrant lower-limb kinematics [138,139]. However, a paucity of research exists outside of ankle proprioception in relation to ankle dynamic stability, revealing a potential gap in the contemporary literature [137].

Proprioception deficits of the trunk are postulated to impair the dynamic stability of the knee, facilitating valgus torque [114,140]. A 3-year prospective cohort study found that females with impairments in trunk neuromuscular control (i.e., lateral displacement, active proprioception repositioning error, and history of low back pain) had a statistically significant increase in subsequent knee injury, likely secondary to an increased propensity for valgus collapse [140]. However, a systematic review by Chia and colleagues [141] found limited support for a causal relationship between trunk proprioception and knee injuries. However, they noted support for increased risk of future knee injuries associated with the combination of an increased knee abduction angle and impaired ipsilateral trunk control [141]. Similarly, for the trunk, the current evidence base would benefit from a greater number of studies investigating proprioception of more proximal joints (e.g., hip and trunk) to elucidate potential avenues for intervention. A paucity of evidence exists regarding both the trunk and hip regarding proprioception in the context of movement performance.

Proprioceptive deficits in athletes landing with DGV may contribute to postural sway, and postural sway has direct implications on movement performance. The movement pattern associated with reduced attenuation of forces upon landing (e.g., reduced hip flexion and reduced knee flexion at initial contact) is likely more prevalent in females secondary to a center of mass posterior to their base of support and may correspond to increased lateral trunk angles upon landing, which may hinder subsequent acceleration, jumping, or changes in direction secondary to a longer time taken for stabilization [26,27,142,143]. These findings are not necessarily seen in male athletes, consistent with much of the research regarding DGV [27]. Nevertheless, neuromuscular control is paramount for optimal knee stabilization upon landing for efficient shock absorption, and impairments in control can significantly hinder the dynamic balance needed for subsequent activity [144]. Athletes with lower centers of mass and larger bases of support upon landing have improved postural stability, supporting that upright and single-leg landings challenge functional stability [47,142,145]. With applied perturbations seen in soccer, rugby, and more, athletes may have increased challenges to reactive postural stability that may further hinder subsequent movement performance [146]. Mediolateral postural sway upon landing may hinder an athlete’s ability to rapidly develop force in an anterior direction. Additional contemporary literature suggests that external auditory and visual stimuli may be barriers to postural stabilization upon landing and facilitators of DGV [34,35,36,37,38].

### 5.3. Dual Task and External Stimuli on Motor Control

Competitive environments comprise a plethora of external stimuli (e.g., auditory and visual) and cognitive demands that may alter lower-limb kinematics upon landing, resulting in changes in efficiency to subsequent motor patterns [34,35,36,37,38]. Zamankhanpour and colleagues [38] found that the inclusion of a cognitive task upon landing among a DGV and a non-DGV group resulted in significantly reduced hip and knee flexion angles in both groups, reducing force attenuation. However, the DGV group had a marked increase in knee abduction angle versus the non-DGV group, with the authors postulating that additional cognitive tasks may challenge motor control and facilitate more risky lower-limb kinematics that increase injury risk and impair subsequent movement performance out of a landing position [38]. This conclusion is broadly supported by the contemporary literature, suggesting that practice in specific environments that mimic the competitive setting may be more beneficial to facilitate optimal motor coordination [34,35,36,37]. For instance, combining cognitive challenges with cutting maneuvers has been found to result in reduced force attenuation (e.g., less knee flexion) and greater DGV among female athletes [34]. Similarly, a study investigating both males and females found that cutting maneuvers while having to pay attention to a ball resulted in a marked reduction in hip flexion and knee flexion and an increase in knee abduction versus a male group [36]. Therefore, these findings may support other literature suggesting that female athletes are more likely to show increased knee abduction angles, likely secondary to gluteal strength and motor control differences versus males [27,36,58,59,93]. The studies above are largely in the context of injury, but the reductions in SSC efficiency (thereby affecting RFD), increased time to stabilization, increased postural sway, and other mechanisms of reduced motor efficiency seen above may be a disadvantage to acceleration, changes in direction, jumping, and other movements out of a double or single-leg jump [24,26,27,34,35,36,37,38,128,129]. Future research may benefit from assessing whether training with dual cognitive tasks results in a reduction in aberrant lower-limb kinematics in athletic motor patterns.

The above sections emphasize the role of DGV in movement performance. A lack of experimental evidence exists regarding DGV on RFD, propulsion in the context of SSC, variability of landing mechanics during sport secondary to proprioception, the implications of multi-joint proprioception and RFD post landing, the relationship between landing patterns and resistance to perturbation, and the potential benefits of dual task training on optimizing landing mechanics. These gaps are highlighted below in Table 2.

## 6. Conclusions

Much of the research regarding DGV appears to exist in the context of injury. Therefore, this review elucidates performance implications associated with DGV within the context of regional interdependency and systems theory, understanding the biomechanical and psychosocial contributors to altered landing mechanics and subsequent athletic movement performance. Females appear to have increased susceptibility to DGV upon landing, likely associated with the significant increase in injury versus males [7,19,21,22,27,58,59,61,68,92,118,142]. DGV may challenge subsequent movement optimization secondary to an impaired time to stabilization and RFD out of the landing phase, alongside challenging reactive postural stability [22,26,27,130,146]. Clinicians, rehabilitation specialists, and strength and conditioning professionals may benefit from emphasizing landing mechanics from the context of regional interdependency and the potential implications of external stimuli and dual task on motor control [4,5,7,9,14,25,26,27,28,29,34,35,36,37,38,41,42,43,54,55]. Contemporary research suggests that significant strengthening of the dynamic stabilizers of the knee against DGV (e.g., hip abductors) may reduce medial knee displacement upon landing and improve force attenuation, and specific practice of the motor patterns required in sport (e.g., acceleration out of a vertical jump) may be beneficial for optimizing motor control, improving performance, and safety [1,2,10,32,78]. However, the presence of additional cognitive tasks may alter lower-limb kinematics; cognitive challenges may be emphasized in training to best mimic the competitive environment, limiting variability in motor performance in the presence of dual tasks [34,35,36,37,38]. This review provides novel information through investigating multi-joint muscular contributions to DGV upon landing on performance variables (e.g., RFD, balance, dual task), suggesting that excessive valgus torque at the knee may hinder subsequent movement performance [4,7,9,22,25,27,28,29,34,35,36,37,38,41,118,119,130,142]. Research regarding sport performance would benefit from investigating excessive DGV post landing on subsequent acceleration, jumping, change-of-direction, and perturbation response to better elucidate practical implications of aberrant frontal plane knee kinematics upon landing. These outcomes may benefit from being coupled with dual task or other external stimuli (e.g., crowd noise) for improved applicability to competitive performance. Secondary to the existing research on DGV that notes variation between males and females, future research should continue to consider these differences in the context of movement performance [7,19,21,22,27,58,59,61,68,92,118,142]. Addressing these gaps in the contemporary literature may guide both rehabilitative and training programs for athletes.

## Figures and Tables

**Table 1:** Future research opportunities in lower-limb biomechanics in the context of landing efficiency.

Research Gaps	
1	Gluteal, quadriceps, and plantarflexor force generation in various landing strategies for subsequent sprinting
2	Semitendinosus strength or pre-activation with DGV in single-leg landing tasks for improved modeling of force attenuation
3	Subtalar joint responses to single-leg landing to improve the contemporary understanding of dynamic force coupling at the level of the foot and ankle
4	Proprioceptive and kinesthetic sense of the ankle in relation to more superior joint mechanics during landing
5	Trunk motor control (e.g., co-contraction ratios of left versus right lateral flexors and rotators) during landing on DGV and its relationship to subsequent force production
6	Investigation into the quadratus lumborum and gluteus medius force couple on landing mechanics

**Table 2:** Future research opportunities on performance implications of DGV.

Research Gaps	
1	Landing mechanics and subsequent multiplanar RFD for efficiency in reactive stability in sport
2	The relationship between DGV and propulsion in the context of the SSC
3	Foot and ankle proprioception compared to variability of landing mechanics (e.g., foot positioning)
4	Multi-joint proprioception (e.g., including the trunk, hip, and knee) and RFD post landing
5	Applied perturbations and knee frontal plane mechanics in trained versus untrained athletes for insight into reactive postural stability and DGV
6	Dual task training on ameliorating DGV in athletes

## Data Availability

No new data were created or analyzed in this study. Data sharing is not applicable to this article.
